# Neural correlates of unconscious processing in functional magnetic resonance imaging: does brain activity contain more information than can be consciously reported?

**DOI:** 10.1093/nc/niaf042

**Published:** 2025-11-11

**Authors:** Joaquim Streicher, Sascha Meyen, Volker H Franz, Timo Stein

**Affiliations:** Département de neurosciences, Université de Montréal, Pavillon Paul-G.-Desmarais, 2960, chemin de la Tour, local 111, Montreal, Québec H3T 1J4, Canada; Center for Advanced Research in Sleep Medicine, Hôpital du Sacré-Cœur de Montréal, CIUSSS du Nord-de-l’île-de-Montréal, 5400 Boulevard Gouin Ouest, 5th floor, Wing J, Door 5080, Montréal, Québec H4J 1C5, Canada; Integrated Trauma Center, Hôpital du Sacré-Cœur de Montréal, CIUSSS du Nord-de-l’île-de-Montréal, 5400, boulevard Gouin Ouest, Montréal, Québec H4J 1C5, Canada; Department of Computer Science, University of Tübingen, Sand 14, 72076 Tübingen, Germany; Department of Computer Science, University of Tübingen, Sand 14, 72076 Tübingen, Germany; Department of Psychology, University of Amsterdam, Nieuwe Achtergracht 129-B, 1018 WT Amsterdam, The Netherlands

**Keywords:** neural correlates of consciousness, fMRI, reanalysis, indirect task advantage, signal detection theory

## Abstract

A central question of consciousness research is which cognitive processes can occur unconsciously. To investigate this, researchers typically compare participants’ ability to consciously discriminate a stimulus to their unconscious processing of the same stimulus (e.g. measured via reaction time or brain activity). If participants are not significantly different from chance in the awareness (or “direct”) measure while nevertheless there is a significant effect in the processing (or “indirect”) measure, researchers argue that there is no conscious processing of the stimulus, while the stimulus is nevertheless somehow processed, as indicated by the processing measure. In consequence researchers conclude that the stimulus has been processed unconsciously. Using neuroimaging techniques such as functional magnetic resonance imaging (fMRI), researchers then infer which brain regions are involved in unconscious versus conscious processing. However, this methodology is based on a fundamental statistical fallacy that has likely led to an overestimation of the scope of unconscious processing, regarding both its capacity and the brain areas involved. The key problem is that sensitivities in the two measures are never directly compared. Therefore, it is not appropriate to conclude that the processing measure had higher sensitivity than the awareness measure. We reanalyzed the results from 16 fMRI studies directly comparing the sensitivities of both measures in 80 experimental conditions. Our results show that, using this sensitivity comparison method, only eight experimental conditions provide evidence for unconscious processing. These results question the validity of the interpretations commonly drawn in the field.

## Introduction

Consciousness is currently one of the most fascinating and challenging topics in cognitive neuroscience and related fields (Seth 2018, Michel et al. 2019, Dehaene et al. 2021). In the last decades, consciousness research has been focused on investigating brain activity associated with conscious processing, or neural correlates of consciousness (NCCs; Crick and Koch 1990, Koch et al. 2016, Rees et al. 2002). In this quest, functional magnetic resonance imaging (fMRI) has been and continues to be one of the most widely used neuroimaging methods, and fMRI studies constitute an important milestone in the consciousness science landscape (Hesselmann 2013). A common approach to investigate NCCs is to test the scope of unconscious processing (Kouider and Dehaene 2007, Lin and He 2008).

### The standard reasoning to investigate unconscious processing

A widely used reasoning to isolate unconscious processing is to collect two measures: a measure of stimulus awareness, or *direct task*—e.g. visibility ratings or discrimination responses in a task with a binary response format—and a measure of stimulus processing, or *indirect task*—e.g. reaction time (RT) or brain activity (Hannula et al. 2005, Schmidt and Vorberg 2006, Simons et al. 2007, Sand and Nilsson 2016, Shanks et al. 2021). Note that here we employ the term “indirect task” for consistency with the behavioral priming literature, but that it could be used interchangeably with “indirect measure” or “processing measure,” terms that may be more appropriate in the case of neuroimaging studies where participants often do not perform a separate task but indirect measures are taken in parallel. Similarly, the “direct task” is sometimes referred to as “direct measure” or “awareness measure.”

**Figure 1:**
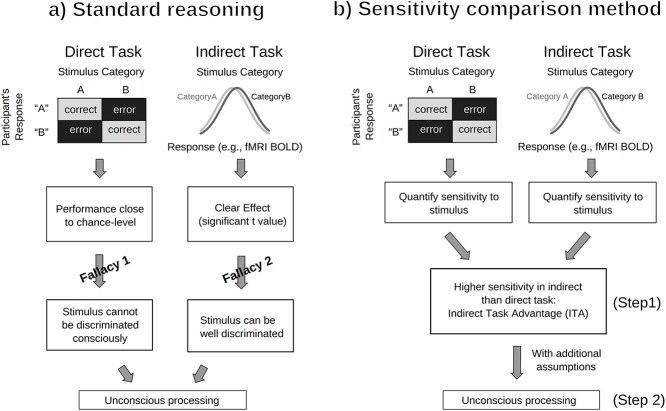
Inferring unconscious processing using neuroimaging: standard reasoning versus sensitivity comparison. *Note*. Demonstrating an ITA—higher sensitivity in indirect than direct responses—is a prerequisite for the typical, further-reaching inferences about conscious/unconscious processing. (a) In the standard reasoning, or double *t*-test approach, the direct task is performed to ensure participants’ unawareness and any effect obtained through the indirect task serves as evidence that brain data is sensitive to stimulus information. However, there are two fundamental statistical fallacies here. Fallacy 1: the absence of a significant effect for the direct task cannot constitute evidence for the absence of awareness, especially as the direct task is often underpowered. Fallacy 2: a significant effect for the indirect task does not necessarily imply good sensitivity. (b) The appropriate analysis requires a sensitivity comparison and thus an explicit calculation of the indirect task’s sensitivity. The sensitivities of the two measures can then be compared to determine if there is an ITA or not (Step 1). If an ITA is confirmed, one must justify that the observed ITA is attributable to unconscious processing rather than some other phenomenon (Step 2). Adapted from Meyen et al. (2022), used under the Creative Commons Attribution 4.0 International License.

If participants perform at chance when responding to the stimulus directly (typically tested by performing a *t*-test), any effect obtained through the indirect task is considered evidence that the stimulus was processed unconsciously (Stockart et al. 2024). This approach is sometimes referred to as the “double *t*-test” approach, as it consists in performing an initial *t*-test to assess participants’ awareness, followed by a second t-test to evaluate unconscious processing (Fig. 1a).

When the indirect task is performed using neuroimaging techniques such as fMRI, significant changes of brain activity or decoding performance serve as evidence that the stimulus was processed by the brain, whereas the initial awareness *t*-test reveals whether participants were able to discriminate the stimulus better than chance or not. One can then allegedly infer which brain regions are involved in the unconscious processing of the stimulus and, by comparison, deduce which brain regions are involved in its conscious processing (i.e. NCCs). Following that methodology, numerous stimuli with different level of cognitive complexity have been suggested to be processed unconsciously: Gabor patches and gratings, objects and tools, words and semantic objects, emotional faces, or body postures (Dehaene et al. 2001, Haynes and Rees 2005, Dannlowski et al. 2007, Hesselmann and Malach 2011, Prochnow et al. 2013, Imamoglu et al. 2014, Ulrich et al. 2014, Suslow et al. 2015, Torralbo et al. 2016, Tettamanti et al. 2017, Zhan et al. 2018, Sheikh et al. 2019). Critically, many studies suggest that high-level cognitive functions can operate without consciousness: memory, language abilities, high-level social skills, or cognitive control (Marois et al. 2004, Kouider et al. 2007, Van Gaal et al. 2010, Freeman et al. 2014, Axelrod et al. 2015, Züst et al. 2015, Rosenthal et al. 2016). The accumulation of results supporting the existence of unconscious perception and cognition in the last decades has shaped our current understanding of unconscious processing and the brain networks it involves, and by extension our definition of the NCCs. But there is a problem with this approach.

### Fallacy in the double *t*-test approach

The limits of unconscious processing are highly debated, and a major consideration when testing those limits is the proper manipulation and assessment of participants’ (lack of) awareness (Reingold and Merikle 1990, Schmidt and Vorberg 2006, Stein and Peelen 2021). This is not trivial, and there exist many pitfalls for consciousness researchers: invalid assessment of awareness, biased experimental design, or lack of statistical power (Newell and Shanks 2014, Vadillo et al. 2020, 2022, Stein et al. 2021, 2024, Stein and Peelen 2021, Phillips 2021a,b). Crucially, the standard reasoning to rule out consciousness has been shown to be insufficient to conclude the existence of unconscious processing because it lacks the fundamental statistical test that would allow researchers to conclude that the indirect task outperforms the direct task: the measures of the two tasks are never actually compared (Eriksen 1960, Reingold and Merikle 1988, Schmidt and Vorberg 2006, Meyen et al. 2022).

In the framework of the standard reasoning, researchers rule out awareness when participants are not significantly different from chance when discriminating the stimuli (direct measure), and then assume that significant effects in the indirect measures (e.g. brain activity) were reflecting unconscious processing (cf. Fig. 1a). However, there are two major problems with this reasoning: (i) The finding that the direct task is not significantly different from chance is not sufficient evidence for the absence of sensitivity (Fallacy 1 in Fig. 1a). To make things worse, the direct task is often severely underpowered and therefore likely to yield false negative results (Vadillo et al. 2016, 2020). (ii) A significant effect in the indirect task is not sufficient evidence to conclude that the indirect task showed relatively good sensitivity to the stimulus—better than the sensitivity in the direct task (Fallacy 2 in Fig. 1a). Quite to the contrary, it has been shown repeatedly that in studies using the standard reasoning, the sensitivity of the indirect task was just as poor as that of the direct task (Franz et al. 2024, Franz and von Luxburg 2015, Meyen et al. 2022, 2024, Schnepf et al. 2022, Zerweck et al. 2021). The underlying reason for all these problems is the fact that the procedure of performing two *t*-tests instead of a direct comparison is incorrect *per se* (e.g. Franz and Gegenfurtner 2008/Appendix B, Gelman and Stern 2006, Nieuwenhuis et al. 2011; similar problems also exist for Bayesian analyses, Palfi and Dienes 2020). All in all, without a statistical comparison of the sensitivities of the two tasks, one cannot conclude that the indirect task was more sensitive to stimulus information than the direct task, and therefore it cannot follow that there is evidence for unconscious processing.

### The sensitivity comparison method

To replace the double *t*-test approach of the standard reasoning with a more appropriate approach, an alternative methodology has recently been advocated (Meyen et al. 2022, 2024, Zerweck et al. 2021) which is consistent with previous recommendations (Reingold and Merikle 1988, Schmidt and Vorberg 2006). The goal of this *sensitivity comparison method* (Fig. 1b) is to directly compare the performances in the indirect and direct tasks. To achieve this, the sensitivity to the stimulus is calculated for each task (*d*′ values). This allows testing whether the *sensitivity of the indirect task really outperforms the sensitivity of the direct task*, a situation that was dubbed indirect task advantage (ITA, Meyen et al. 2022).

Note that establishing an ITA is a necessary condition for all the inferences about conscious and unconscious processing that are typically drawn (this purely empirical condition is therefore called Step 1 in Fig. 1b). Only if an ITA is established, we can then go on to draw further-reaching inferences about consciousness and NCCs (Step 2 in Fig. 1b). For example, in this second step, one would need to assess whether the direct task adequately measured consciousness and the indirect task adequately measured unconscious processing. For the purposes of the current article, however, it is sufficient to focus exclusively on Step 1: Can we establish an ITA? We will show that the data of many studies do not seem to support this first step on which all the further-reaching inferences are built.

The sensitivity comparison method has already been practically applied to diverse datasets, namely, unconscious priming (Meyen et al. 2022), unconscious number processing (Zerweck et al. 2021), unconscious contextual cueing (Meyen et al. 2024), unconscious response inhibition (Huang et al. 2023), and event-related potentials analysis in the context of unconscious perception (Schnepf et al. 2022).

### The sensitivity comparison method applied to fMRI studies

In this study, we extended the sensitivity comparison method to neuroimaging studies and reanalyzed the results of 16 fMRI studies. Our objective was twofold: investigating the validity of previous interpretations supporting unconscious processing in fMRI studies, and therefore their impact on our definition of the NCCs, and demonstrating that a systematic use of the sensitivity comparison method would help future studies to overcome most of the biases currently undermining the field of consciousness research. We did not apply the sensitivity comparison method to the behavioral results of these studies because we wanted to focus solely on the fMRI results (note, however, that the behavioral results of some of those studies were already analyzed in Meyen et al. 2022).

We used a conservative *benefit-of-the-doubt approach* when reanalyzing the results from the selected studies, meaning that our methodological choices always gave an advantage to the indirect task over the direct task, and therefore favored finding an ITA (cf. Meyen et al. 2022). Because we found little evidence for ITAs, this benefit-of-the-doubt approach makes the case even stronger (“although we favored the indirect task, its sensitivity was not larger than that of the direct task”). In a similar vein, we also did not question the validity of the measures employed. That is, we fully accepted the original studies’ assumptions that measures matched the intended constructs (e.g. that the direct task measured conscious processing and the indirect task unconscious processing). For example, if the stimulus feature at test in the direct task did not correspond to the feature at test in the indirect task, we still included the study, although the indirect task did not reflect processing of the feature of interest (for a detailed discussion of such problems, see Schmidt and Biafora 2023). Thus, we conducted a purely empirical reanalysis of the data allegedly supporting an ITA (Step 1 in Fig. 1b) and did not assess the validity of attributing an ITA to unconscious processing (Step 2 in Fig. 1b, more on this in the *Discussion* section).

## Materials and methods

### Selection process

**Figure 2:**
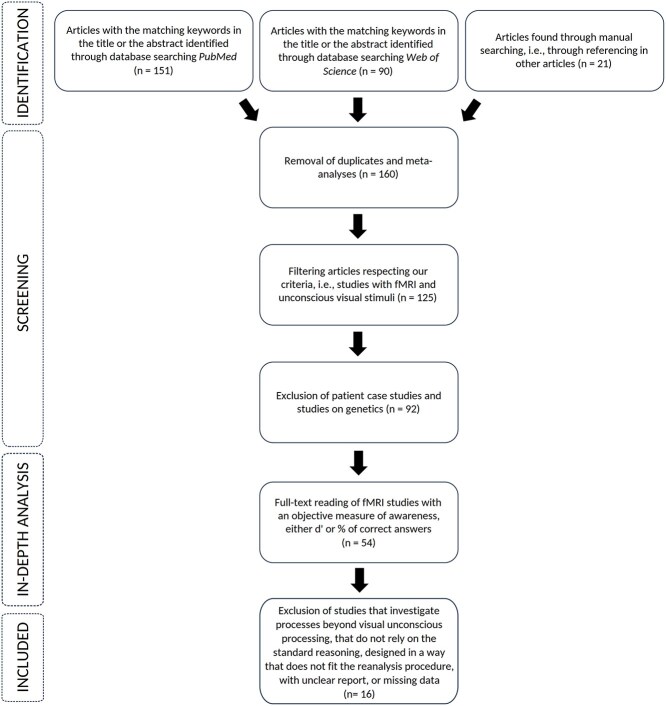
Selection of the studies for the reanalysis. *Note*. Pipeline for selecting studies for reanalysis. The number of studies remaining after each of the exclusion steps is reported in the figure (n). *Identification*. We used the two databases PubMed and web of science and a manual search to constitute a first corpus. Keywords are available in the supplementary material. *Screening*. We went through the titles and abstracts to remove duplicates, meta-analyses, and articles not fulfilling our main criteria. *In-depth analysis*. Full-text articles from studies with an objective measure of awareness were analyzed. *Included*. Sixteen studies were finally selected for the reanalysis. We excluded studies investigating processes beyond visual unconscious processing and visual perception (e.g. working memory or implicit learning), studies that did not solely base their conclusions on the standard reasoning, studies with a design not allowing us to perform the reanalysis (e.g. correlation analysis), studies from which the design or results were ambiguous, or with missing data necessary for the reanalysis.

To select the studies for the reanalysis, we went through a bottleneck selection process (Fig. 2). We first searched for all fMRI consciousness studies investigating unconscious processing of visual stimuli using two databases (PubMed and Web of Science)—keywords are available in the supplementary material section. Duplicates and meta-analyses were excluded right away, either via the databases’ selection criteria or by a manual screening of the articles’ titles and abstracts. A small proportion of the articles were found through manual searching, meaning that they were cited in articles we found through the database searching. The filtering tools offered by the two databases partly failed to isolate the studies of interest, and we went through the articles’ abstracts to exclude studies not meeting our criteria (no fMRI, no method for rendering a stimulus unconscious). We also decided to exclude patient studies from our corpus, although reanalyzing data from those studies could be interesting in the future. We next read through all the remaining articles to only keep studies with an “objective” measure of awareness (i.e. one that quantified behavioral performance with regard to an objectively measurable stimulus property, as opposed to “subjective” measures that rely on participant’s unfalsifiable introspective reports such as “seen”/“unseen” judgments), as this was required to perform the reanalysis.

Studies fulfilling all the above-mentioned criteria were analyzed in depth and cut down to 16 studies (Axelrod et al. 2015, Dehaene et al. 2001, Fang et al. 2005, Fogelson et al. 2014, Freeman et al. 2014, Haynes & Rees 2005, Kouider et al. 2007, Kouider et al. 2009, Kouider et al. 2016, Moutoussis & Zeki 2002, Schurger et al. 2010, Stein et al. 2021, Sterzer et al. 2008, Ulrich & Kiefer 2016, van Gaal et al. 2010, Yang et al. 2012) based on five criteria: First, we focused our reanalysis on visual unconscious processing, and studies investigating other processes such as working memory, implicit learning, or negative compatibility effects were excluded. Based on this criterion, we excluded *n* = 12 studies (note that some studies were excluded based on more than one criterion). Second, studies had to claim unconscious processing following the standard reasoning. Studies that used other rationales or methods (e.g. correlation analysis between the awareness assessment measure and brain activity), were excluded. We excluded *n* = 13 additional studies based on this criterion. Third, studies had to present a design that allowed the application of our reanalysis method. Studies that had a behavioral measure that differed from the traditional binary response format (e.g. a direct task with four response alternatives), or that presented fMRI analysis beyond univariate or multivariate analysis (e.g. functional connectivity analysis) were excluded (*n* = 5 additional exclusions). Fourth, the designs of the selected studies had to allow for a comparison between direct and indirect tasks, and values had to be reported unambiguously. Studies designed in a way that allowed reanalysis but made conclusions difficult to interpret (e.g. because there was an important conceptual mismatch between the stimulus feature tested in the direct task and the stimulus feature tested in the indirect task) were excluded (*n* = 3 additional studies excluded). Fifth, studies had to report enough data to allow for our reanalysis (e.g. *d*′, number of trials, etc.). We contacted the authors of seven studies that otherwise fulfilled all our inclusion criteria (three studies failed also other criteria such that we did not contact the authors). We received only one reply. Unfortunately, such low return-rates are to be expected (Wicherts et al. 2006). After further deliberation, we managed to reanalyze results from one of these studies based on the summary statistics available online. We therefore excluded *n* = 5 additional studies based on this criterion.

Importantly, many of the studies finally selected for the reanalysis presented methodological issues (participant and trial exclusions, inconsistent feature of discrimination, separate task for the direct measure) that probably had an impact on the results of our reanalysis. However, it is important to note that, in agreement with our benefit-of-the-doubt approach, these methodological caveats always biased our reanalysis results toward finding evidence for an ITA in favor of unconscious processing (see *Discussion* section). Note also that we reanalyzed the most promising results from the 16 selected studies, i.e. significant ROIs or results used by the authors to demonstrate unconscious processing. These 80 experimental conditions had the largest effects and therefore highest indirect sensitivities. We did not reanalyze results that, *a priori*, were not expected to yield ITAs, and we did not conduct additional analyses of alternative candidate ROIs to avoid strawman arguments. However, different ROIs may potentially yield more ITAs in future reanalyses.

### The sensitivity comparison method applied to fMRI data

The sensitivity comparison method (Meyen et al. 2022) consists in estimating a sensitivity value (here *d*′) for the indirect task, such that this sensitivity can be compared to the sensitivity of the direct task (which is typically reported in studies). This makes it possible to determine whether there is an ITA (i.e. whether the sensitivity in the indirect task is superior to the sensitivity in the direct task). When using this method to reanalyze existing studies, one is faced with one problem, though: Typically, one would need the trial-by-trial data to estimate the sensitivity. Because those data are often not available anymore, Meyen et al. (2022) developed a method to estimate the sensitivities from the typically published summary statistics (e.g. the results of a *t*-test). This method requires only one additional parameter *q*^2^, which corresponds to the ratio of between-subject to within-subject variability. As such, it is independent of the number of participants and trials, *N* and *K*, which is particularly useful when reanalyzing studies with different *N* and *K*. Practically, *q*^2^ can be thought of as a single-trial reliability. It can be mathematically shown to be equal to the variance of participants’ underlying individual sensitivities. This parameter *q*^2^ can be estimated from similar studies for which trial-by-trial data are available. Here we used the data from Stein et al. (2021) to estimate this parameter for fMRI data and then used this estimate to reanalyze the selected studies. Stein et al. (2021) was selected to determine *q*^2^ as it was the only study for which we had access to a complete dataset, but also because it seemed to provide us with a representative estimate: This study uses stimuli that are commonly used in research on unconscious perception, pictures of faces and houses. Moreover, the sample size was high (*N* = 43) and, compared to the other studies that were selected for our reanalysis, this study presented fewer of the confounding biases we mentioned above.

To estimate the parameter *q*^2^, we used each participant’s observed, individual sensitivity that Stein et al. (2021) had obtained by decoding fMRI data of the lateral occipital cortex (LOC). We chose the LOC results because they seemed most promising to show an ITA. We conducted a Bayesian analysis to determine the highest posterior density interval (Hyndman 1996; Kruschke 2021) for *q*^2^. Starting with a Jeffreys’ prior on *q*^2^, we employed a Markov Chain Monte Carlo algorithm because it can efficiently estimate posterior distributions with multiple variables making optimal use of each individual sensitivity instead of just the observed standard deviation. We found a posterior mean *q*^2^ = 0.0061 (*q* = 0.08) with 95% HDI = [0.001, 0.019]. Note that a simpler, naïve plugin estimation consistently yielded a mean *q*^2^ = 0.0059. This ratio for fMRI data is somewhat smaller than what we had estimated for RT data (with a conservative assumption, we had *q*^2^ = 0.0225 there). Again, this decrease is plausible due to fMRI measurements incurring more trial-by-trial noise. Thus, we used for all our subsequent reanalyses of fMRI data the parameter estimate *q*^2^ = 0.006. This corresponds to assuming that the standard deviation of individual, underlying sensitivities is *q* = 0.08 (observed sensitivities may vary more due to sampling noise). We also estimated *q*^2^ based on the other conditions of Stein et al. (2021), namely fMRI measures at different time points during the trials (at 0, 1.6, 3.2, 4.8, 6.4, and 8.0 s after stimulus onset). Estimates of *q*^2^ in the objectively invisible condition were on average *q*^2^ = 0.005 and in the subjectively invisible condition *q*^2^ = 0.004 (see Supplementary Fig. S1). The largest estimate we found in any of the conditions was *q*^2^ = 0.008, which is likely an overestimate. Had we chosen this value instead of *q*^2^ = 0.006 for our reanalyses, the estimated indirect task sensitivities would only change marginally (e.g. from *d*′ = 0.147 to *d*′ = 0.152 in the first reanalyzed condition of Dehaene et al. 2001).

With this new *q*^2^, we applied the sensitivity comparison method to the 16 fMRI consciousness studies that were selected for our reanalysis by estimating *d*′ from published summary statistics given minimal assumptions. The following formula summarizes how *d*′ was estimated for the indirect task:


(1) \begin{equation*} {d}_{\mathrm{indirect}}^{\prime }=t\cdot{c}_{N,K,q{}^2} \end{equation*}

where the *d*′ value for the indirect task—here parameter estimates from a general linear model fitted to the fMRI data or multivariate pattern analysis (MVPA) values—is estimated based on the reported *t*-value of a paired *t*-test comparing two conditions with different neural activation. The constant ${c}_{N,K,q{}^2}$ adjusts for the increase in t values that occurs with a greater number of participants (*N*), a higher number of trials (*K*), and it accounts for the dependency on the ratio of between-subject to within-subject variance (*q*^2^). This scaling factor ${c}_{N,K,q{}^2}$ was computed for each reanalyzed study and condition separately, where we used the respective *N* and *K* but always the same *q*^2^ = 0.006. When *t* values were missing, we transformed *z* scores using quantile mapping: We first computed the associated *p*-value and then the associated t value based on the number of degrees of freedom. When values were not clearly reported or when there seemed to be ambiguity, we always selected the value giving an advantage to the unconscious processing conclusion, in order to follow our benefit-of-the-doubt approach. Analyses can be performed by using our website (http://www.ecogsci.cs.uni-tuebingen.de/ITAcalculator/) or, for more flexibility, the R code available on OSF (https://osf.io/wnfta/).

## Results

**Figure 3:**
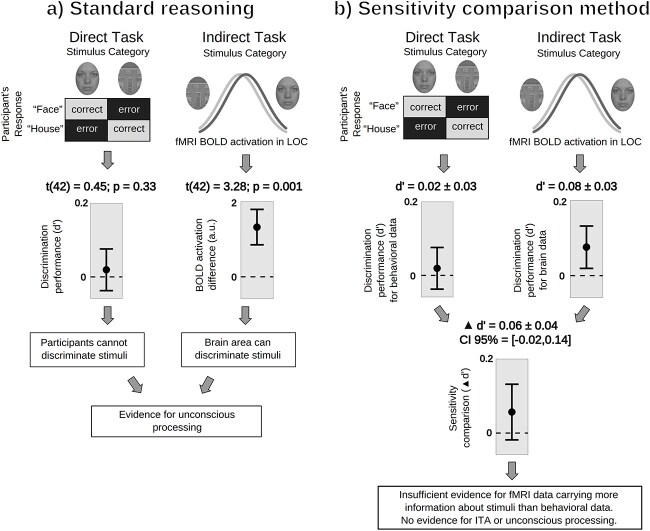
Standard reasoning versus sensitivity comparison method: Example data from Stein et al. (2021). *Note*. Example of the two procedures in Stein et al. (2021) for the univariate analysis of the LOC. (a) Erroneous double t-test approach (“standard reasoning”): Consciousness researchers usually test participants’ ability to discriminate stimuli, here faces and houses, by computing a *d*′ value and testing it against zero. If *d*′ is not significantly different from zero (*P* > .05), participants are considered unaware of the stimuli’s features of interest. On the other hand, a significant difference in brain activity, here BOLD variations in LOC, is then considered as evidence that the stimuli’s features of interest are processed unconsciously. This standard reasoning is, however, flawed (cf. Fig. 1). (b) Appropriate sensitivity comparison method: A *d*′ value is computed for both behavioral and brain measures and the two values are directly compared. Here there is no significant difference between the two sensitivity values, suggesting that there is insufficient evidence for brain data being more sensitive to stimulus information than behavioral data. Note that the sensitivity for the indirect task is significantly above chance, but that this test alone is not enough to conclude that the sensitivity of the indirect task outperforms the sensitivity of the direct task. Brain data should contain more information relative to the stimulus than behavioral data for one to conclude that there is an ITA and, potentially, evidence for unconscious processing. Error bars depict 95% CIs. The BOLD activation plot in the top row is only for illustrative purposes and not based on data.

**Figure 4:**
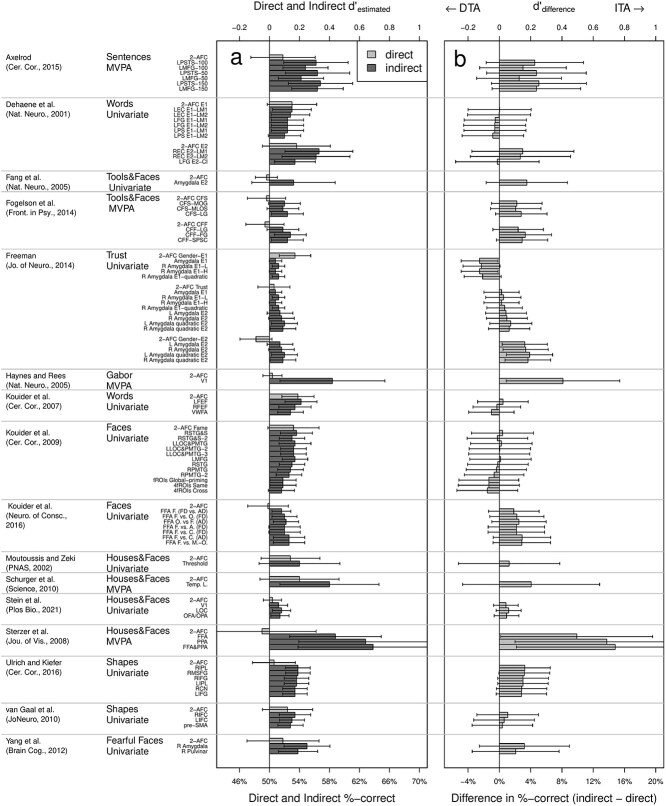
Sensitivity comparison in 16 fMRI studies investigating unconscious processing. *Note*. (a) Sensitivity for the direct and indirect tasks. (b) Sensitivity comparison between the two tasks. The difference between the sensitivities of the two tasks is reported on the horizontal axis. 95% CIs are reported using error bars. The data used to perform the reanalysis is available in Supplementary Tables S1–S16. DTA is short for direct task advantage, i.e. when there is evidence that the direct task outperforms the indirect task. Acronyms and abbreviations from the different conditions are available in the supplementary material.

We first provide an example by applying the sensitivity comparison method to data from Stein et al. (2021)—see Fig. 3. We then give a summary of all results from our reanalysis (Fig. 4).

### The sensitivity comparison method applied to Stein et al. (2021)

In the study of Stein et al. (2021), 43 participants were presented with masked house and face stimuli (200 trials per participant). Participants discriminated the presented stimulus in each trial (direct task). Stein and colleagues reported a *d*′ of 0.02 (SE = 0.038) for the direct task and *t*(42) = 3.28 for MVPA decoding of faces versus houses in LOC (highest reported value for the indirect task) and—based on the standard reasoning—infer an ITA.

From this published *t*-value, we estimated for the indirect task a sensitivity of *d*′ = 0.08 (SE = 0.03). This resulted in a *d*′ difference between indirect and direct tasks of 0.06 (SE = 0.04), with a 95% confidence interval (CI) including zero (95% CI = [−0.02, 0.14]), thereby indicating that the sensitivity-difference did not deviate significantly from zero. That is, although the sensitivity of the indirect task was slightly larger than that of the direct task, this small difference can be explained by noise: There was not enough evidence to support an ITA based on the reported statistics. These results are depicted in detail in Fig. 3 and in a more compact fashion in Fig. 4. This is the analysis we applied to all included studies (see below).

To demonstrate that the sensitivity comparison method is in fact capable of detecting ITAs, we conducted an additional analysis where we contrasted conditions of Stein et al. (2021) for which it was *a priori* plausible to expect a difference. For this, we compared the LOC activity in the “objectively *visible* condition” (*t* = 16.69; *d*′ = 0.4; SE = 0.05) with the direct task performance of the “objectively *invisible* condition” (*d*′ = 0.02, SE = 0.04). As a result, we found a difference of 0.38 (SE = 0.06; 95% CI = [0.26; 0.50]). This shows that our method is *in principle* able to detect an ITA: If processing is sufficiently strong in the indirect task, sensitivity differences can be detected. But note that, in the “objectively visible condition,” participants’ direct task sensitivity was also higher with *d*′ = 4.5 so that no evidence for unconscious processing is given by this comparison either.

### The sensitivity comparison method applied to all studies

Next, we applied the sensitivity comparison method to all selected studies and depict the results in Fig. 4. Inspection of this figure shows that in most conditions, the sensitivity difference was close to zero and not significantly different from zero (error bars correspond to 95% CIs).

Only eight of the 80 experimental conditions (10%) showed a significant ITA, while two conditions (2.5%) showed a significant opposite effect (dubbed direct task advantage; DTA; in the figure). Note that one would *a priori* expect a false positive rate of 5%, and that we did not correct for multiple hypothesis testing. So, overall, there seems little evidence for an ITA across all studies, although all studies inferred unconscious processing based on their reported results. With that, a parsimonious explanation of most of the data is that weak, residual conscious processing underlies responses. In this majority of the cases, there is not enough evidence for processing with a sensitivity beyond that in participants’ direct (conscious) responses. There was insufficient empirical basis for interpretations about unconscious processing.

Nevertheless, it is instructive to have a closer look at the eight conditions that supported an ITA. They came from three studies (Haynes and Rees 2005, Sterzer et al. 2008, Freeman et al. 2014), including two studies using MVPA as the indirect task (Haynes and Rees 2005, Sterzer et al. 2008). We discuss one of them as an illustration in the following. A description of all reanalyzed studies, the data used for the reanalysis such as reported statistics, number of participants, and number of trials as well as all detailed numeric results are available in the supplementary materials (Supplementary Tables S1–S16).

Sterzer et al. (2008) reported a *d*′ of −0.05 (SE = 0.14) for the direct task based on data from five participants (77 trials per participant) performing a binary discrimination task with face and house stimuli rendered invisible using continuous flash suppression (CFS). Based on the reported statistics from their decoding analysis, we estimated a *d*′ of 0.69 (SE = 0.18) for the indirect task (highest decoding accuracy was found when using both FFA and PPA ROIs data: 63.5%), resulting in a sensitivity difference of 0.74 (SE = 0.23)—this was the highest sensitivity difference we found across all experimental conditions of all studies. The 95% CI excluded zero (CI = [0.11, 1.37]), supporting the existence of an ITA.

## Discussion

Our reanalysis evaluated evidence for unconscious processing and its neural correlates in 16 fMRI studies with 80 experimental conditions. We applied our sensitivity comparison reanalysis method on their summary statistics and thereby replaced the problematic standard reasoning (Fig. 1a) with a more appropriate method (Fig. 1b). In most conditions (87.5% of all conditions), there was no significant difference between sensitivities in indirect and direct tasks, 2.5% of conditions showed higher sensitivity for the direct task, and only 10% of conditions showed a larger sensitivity difference in the indirect task than in the direct task—that is an ITA (Fig. 4). However, results from all these conditions had been considered sufficient evidence for unconscious processing in the original studies. As an ITA is a necessary step for further-reaching inferences about conscious and non-conscious processing, a lack of clear evidence for an ITA constitutes a problem for such inferences.

These results suggest that the evidence for unconscious processing has often been overestimated in consciousness studies using fMRI. This is a similar situation as has been found for behavioral priming (Zerweck et al. 2021, Meyen et al. 2022), priming in EEG (Schnepf et al. 2022), implicit learning (Meyen et al. 2024), and lie detection (Franz and von Luxburg 2015, Franz et al. 2024). Note that some of the here reanalyzed fMRI studies also presented behavioral data. The analysis of these behavioral data (performed in Meyen et al. 2022) is in agreement with our results from reanalyzing the fMRI data here: There is little evidence for ITAs in either measure (Meyen et al. 2022). Without establishing an ITA one cannot infer that there were unconscious processing of a stimulus beyond what participants can consciously report. In other words, based on our results it seems likely that brain activity has often been prematurely associated with unconscious processing, which in turn has consequences for our definition and understanding of the NCCs. In the following we discuss some further, open issues.

### Methodological biases and their impact on ITAs

While our reanalysis did not indicate convincing evidence for an ITA in most studies and conditions, we now want to discuss those few conditions where our reanalysis did find evidence for an ITA. What can we conclude for those conditions? Do these ITAs reflect unconscious processing (Step 2 in Fig. 1b)? Unfortunately, some common methodological flaws might bias consciousness studies toward finding evidence for unconscious processing. For example, there often exists a statistical power asymmetry between the two tasks, the direct task being performed with less participants or trials. In fMRI studies, this asymmetry is typically exacerbated by the high number of data points (voxels) in fMRI as well as by the nature of the data: continuous responses for the brain (e.g. BOLD activation of the amygdala) are often compared to binary responses for the participants (e.g. “fearful” versus “neutral”), thus giving the indirect task an advantage in terms of statistical power compared to the direct task.

Additionally, it is common in fMRI studies to have participants perform the two tasks at different points in time, in different contexts, e.g. inside and outside the scanner, or even to have different participants for the two measures. Although this might not always give a spurious advantage to the indirect task, using dissimilar data collection procedures is problematic if one intends to compare the two tasks. Moreover, participants typically have to respond at a designated time window after stimulus presentation, while the brain is measured at the time of stimulus presentation. This creates another asymmetry: in the direct task, participants are burdened by short-term memory demands because information has to be maintained until report, while the indirect task occurs simultaneously (and sometimes the best time points are selected for reporting results).

Some studies use MVPA, an approach in which the information available in brain activity is used in an optimal way to classify stimuli. Comparing these classifications with participants’ direct task classifications requires some additional considerations: First, training a classifier after all trials have been recorded (offline) grants an advantage over participants in the direct task, who have to give responses in an online fashion—participants have to set internal decision thresholds on the fly, which is more difficult than when the threshold is set *post hoc* based on all trials. Second, determining which features of the stimuli allow for optimal classification may only be learned by participants during the experiment, while a trained classifier can optimally weight the neural activity representing those features *post hoc*. Finally, in all fMRI studies, the exclusion of participants or scans, or the formation of two groups *post hoc*, can lead to regression to the mean effects (Barnett et al. 2005, Schmidt 2015, Shanks 2017).

Three studies which did not pass our criteria for inclusion in the reanalysis deserve to be mentioned here, as they complete the overall picture. One study by Cao et al. (2021) did perform a sensitivity comparison (and was therefore not included because no reanalysis was necessary). The authors investigated unconscious processing of facial identity. They presented two faces to participants—either two actors’ faces or two familiar faces—morphed to different degrees and masked by CFS. Facial identity was decoded from fMRI activity in the right fusiform face area and directly compared to participants’ face identity recognition performance (direct task). The authors found only anecdotal evidence for an ITA (*t*(15) = 1.85, *P* = .042 in a one-tailed test, BF = 2.033; Cao et al. 2021). A replication of these results would be helpful to assess the robustness of this effect and to clarify whether facial identity might be a promising line of future research on unconscious processing. Two other studies investigated the unconscious processing of object-context relations (Faivre et al. 2019) and emotional words (Hoffmann et al. 2015). Both studies followed the standard reasoning. However, both reported null findings and therefore did not even claim unconscious processing (this is why we did not include them in our reanalysis). Taken together, their results also indicate that the evidence for ITAs is scarce.

In our reanalysis, studies with and without confirmed ITA are affected by some of the above-mentioned caveats and may thereby result in a biased comparison between direct and indirect tasks in favor of the latter one. This gives more weight to our results. Although studies were affected by these biases (for which we did not correct) inflating evidence for ITAs, we still found little evidence for ITAs overall.

### The role of statistics in neuroimaging studies investigating unconscious processing

A frequent objection of consciousness researchers to the sensitivity comparison method applied in the present reanalysis is that it were underpowered (Stockart et al. 2024). However, demonstrating unconscious processing based on the results of two tasks logically requires an appropriate comparison. If this comparison then turns out to lack statistical power then we should not return to the problematic standard reasoning. Instead, we must first reevaluate the strength of evidence from previous studies and second aim to increase power in future studies. In many of our reanalyzes, the direct task sensitivity lacked precision (larger direct than indirect error bars in Fig. 4a). But the precision of a comparison between two measures is always limited by the lower precision of the two measures. Thus, the demand for higher power directly translates into increasing the number of participants and trials in the direct task. Note that this improvement would even be relatively cheap, given that we are talking about the behavioral task not the indirect task, where imaging is involved. Another option for future studies is to include continuous measures in the direct task to then compare the information in both tasks based on their continuous responses.

### Limitations of the present work

There are some limitations inherent to the present reanalysis. First, our reanalysis procedure requires objective measures of awareness, and we did not consider the case of subjective awareness measures (Seth et al. 2008, Kiefer et al. 2023). Claims about subjectively-unconscious processing, if established with appropriate subjective measures, are not targeted by our sensitivity comparison critique. Second, the estimate of the parameter *q*^2^ was based on a single fMRI study (Stein et al. 2021), which might not be representative of the whole field. Our study provides a first estimate that might evolve in future studies. However, since Stein et al. (2021) had a comparatively large sample size (*N* = 43), it is likely to constitute an appropriate estimation. Third, fMRI data is complex, and we focused on studies with a traditional design (2-class MVPA, 2-conditions *t* values or *F* values from ANOVAs). Since this is the first time such a reanalysis has been performed with fMRI data, extending to all types of design would require additional adaptations of the reanalysis procedure. Similarly, extensions of the sensitivity comparison method should include direct tasks with more than two response alternatives. Fourth, we decided to focus our reanalysis on results interpreted by the authors of the reanalyzed studies as evidence for unconscious processing. Therefore, we did not reanalyze results that, *a priori*, were not expected to yield ITAs. The specificity of our method could thus be further validated in the future by conducting additional analyses of alternative candidate ROIs. Fifth, our approach only used the reported summary statistics—due to the well-known problems of obtaining raw-data from published studies (Wicherts et al. 2006). Performing a sensitivity comparison based on the individual trial-by-trial data could increase statistical power. Sixth, one may criticize that transforming the continuous indirect task responses into binary predictions for each trial in order to compute sensitivity from hit rates and false alarm rates artificially reduces statistical power. Indeed, dichotomization reduces power. But any comparison is necessarily limited by the weakest comparand: If the direct task is by design based on binary responses, an appropriate comparison must use dichotomized indirect task responses. Our argument is that, if the original studies infer unconscious processing based on contrasting the indirect task results to the direct task data measured on a binary scale, the indirect task data should show a difference when brought to that scale. Seventh, some studies did not test the right feature of discrimination, i.e. brain and behavioral datasets were not tested on the same information, such that comparing their sensitivity can be problematic (Reingold and Merikle 1988, Schmidt and Vorberg 2006, Schmidt and Biafora 2023). We recommend future studies to avoid such direct–indirect mismatch by testing the feature of interest in both the direct and indirect task and follow the criteria we mentioned in the previous section (same number of participants and trials, no *post hoc* exclusion, experimental design as similar as possible) to avoid an unfair comparison between direct and indirect tasks (in line with recent recommendations, Stockart et al. 2024). Although some exclusion criteria are clear-cut (e.g. missing data necessary for the reanalysis), others might seem debatable. For example, we decided to focus on visual unconscious processing but future studies could include studies investigating more complex cognitive processes, such as working memory or implicit learning. We also excluded studies with what we considered too heavy methodological biases or for which values were not clearly reported to avoid obtaining non-interpretable or invalid results from the reanalysis. Future reanalyses might offer complementary findings by reanalyzing different sets of studies based on different selection criteria. Finally, although it seems likely that the estimated *q*^2^ value will be within a reasonable range, it might still vary across laboratories and designs. Future work should estimate *q*^2^ using a diversity of fMRI datasets and designs to further improve the generalizability of our conclusions.

### Future directions: investigating the NCCs using the sensitivity comparison method

Our approach can be seen as providing a focus on the most promising designs and methods: Based on this first attempt of reanalysis of a very limited corpus, it appears that studies using MVPA analysis in visual areas (V1, FFA, PPA) were the most potent in confirming an ITA (stimulus type: faces/houses, gratings; suppression method: CFS, sandwich masking). Future studies that will survive the sensitivity comparison will constitute good candidates to define the extent of unconscious processing and identify neural correlates of consciousness.

Current trends in the field as reported in the UnconTrust database (in prep, https://uncontrustdb.tau.ac.il/) suggest that, although there has been an increase in good practices in recent years, suboptimal methodological choices remain a widespread problem (e.g. post-experiment awareness measure, direct–indirect mismatch). Apart from following the criteria we discussed earlier in this work (see also Rothkirch and Hesselmann 2017, Stein et al. 2024, Stockart et al. 2024) we recommend future consciousness studies using fMRI or other neuroimaging techniques to quantitatively compare brain data to behavioral data directly on the same scale—e.g. with *d*′ values, but other tools could be used to compare the two tasks, for instance tools from information theory (Ince et al. 2017, Mediano et al. 2022, Meyen 2022). A systematic use of the sensitivity comparison method will lead to better practice and improved methodology within consciousness research. For example, it is likely that researchers will be more attentive to having enough statistical power in their measure of stimulus awareness, and that more effort will be made to have the two measures resulting from procedures as similar as possible, so that they are comparable. Having brain activity as an indirect measure makes the comparison of sensitivity less straightforward than with behavioral priming (as in Meyen et al. 2022), and having two different quantitative data types (binomial, univariate or multivariate analyses) on two different scales also makes the comparison difficult. The fact that data type and statistical tests vary between the two measures reinforces the need for a comparison of sensitivity on the same scale. Therefore, it will be important to develop additional methods to directly compare brain and behavioral data (e.g. Ince et al. 2017). As a final note, we stress once again that an ITA alone is not sufficient to conclude unconscious processing but that it only represents a necessary first step. Other aspects of validity must be addressed in a second step to make claims about unconscious processing. Future studies are needed to confirm the results from this first reanalysis, and a systematic meta-analysis using the sensitivity comparison could be informative regarding the types of stimuli that yield an effect supporting unconscious processing, as well as the brain networks involved.

## Conclusions

By showing that a large proportion of the fMRI consciousness studies we reanalyzed fail in providing evidence for an ITA, we call for a methodological update of the field of consciousness science. After Meyen et al. (2022) had shown that behavioral priming effects from many consciousness studies are most likely due to statistical artifacts, we expanded this reasoning to studies using fMRI data as an indirect measure. We argue that, to determine that brain activity reflects unconscious stimulus processing, one should provide evidence that brain data is more sensitive to stimulus information than behavioral data from the awareness assessment. However, based on our results, we argue that brain data rarely outperforms behavioral data. Together with the additional methodological flaws undermining the field, the results from our reanalysis put common interpretations in consciousness research into question. We recommend future studies investigating unconscious processing using fMRI and other neuroimaging techniques to support their claims with a direct comparison (of sensitivities) between brain and behavioral data. Such profound methodological changes in the field would contribute to a better understanding of unconscious processing and would provide us with a more accurate working definition of the neural correlates of consciousness.

## Supplementary Material

JoaquimStreicher_NeuralCorrelatesOfFMRI_SupplementaryMaterial_nonhighlighted

## Data Availability

Full results from the reanalysis are available in the supplementary materials. The analysis code with the data underlying this article is available in a repository on Open Science Framework, at https://osf.io/wnfta/.
