# Supplementary material for: Neural correlates of unconscious processing in functional magnetic resonance imaging: does brain activity contain more information than can be consciously reported?
Source: Neurosci Conscious. 2025 Nov 11;2025(1):niaf042. doi: 10.1093/nc/niaf042 (PMC12604470; doi:10.1093/nc/niaf042)
Supplement: JoaquimStreicher_NeuralCorrelatesOfFMRI_SupplementaryMaterial_nonhighlighted [file joaquimstreicher_neuralcorrelatesoffmri_supplementarymaterial_nonhighlighted.pdf]

Supplementary Material

1 Keywords

1.1 PubMed (first search)

fmri[Title/Abstract] AND (unconscious[Title/Abstract] OR nonconscious[Title/Abstract] OR non-conscious[Title/Abstract] OR invisible[Title/Abstract] OR ‘outside awareness’[Title/Abstract] OR unseen[Title/Abstract] OR ‘without awareness’[Title/Abstract] OR subliminal[Title/Abstract] OR implicit[Title/Abstract]) AND (mask*[Title/Abstract] OR ’binocular rivalry’[Title/Abstract] OR ’binocular suppression’[Title/Abstract] OR ’interocular suppression’[Title/Abstract] OR ’continuous suppression’[Title/Abstract] OR ’flash suppression’[Title/Abstract])

1.2 Web of Science

TI=(fmri AND (unconscious OR nonconscious OR non-conscious OR invisible OR ‘outside awareness’ OR unseen OR ‘without awareness’ OR subliminal OR implicit) AND (mask* OR ’binocular rivalry’ OR ’binocular suppression’ OR ’interocular suppression’ OR ’continuous suppression’ OR ’flash suppression’)) OR AB=(fmri AND (unconscious OR nonconscious OR non-conscious OR invisible OR ‘outside awareness’ OR unseen OR ‘without awareness’ OR subliminal OR implicit) AND (mask* OR ’binocular rivalry’ OR ’binocular suppression’ OR ’interocular suppression’ OR ’continuous suppression’ OR ’flash suppression’)) OR (fmri AND (‘attentional blink’[Title/Abstract] OR ’inattentional blindness’[Title/Abstract] OR ’change blindness’[Title/Abstract])))

1.3 PubMed (second search)

fmri[Title/Abstract] AND (‘attentional blink’[Title/Abstract] OR ’inattentional blindness’[Title/Abstract] OR ’change blindness’[Title/Abstract])

2 Estimation of q^2 from other experimental conditions

Figure S1

Estimates of the Variance Ratio q2 from Stein et al. (2021)

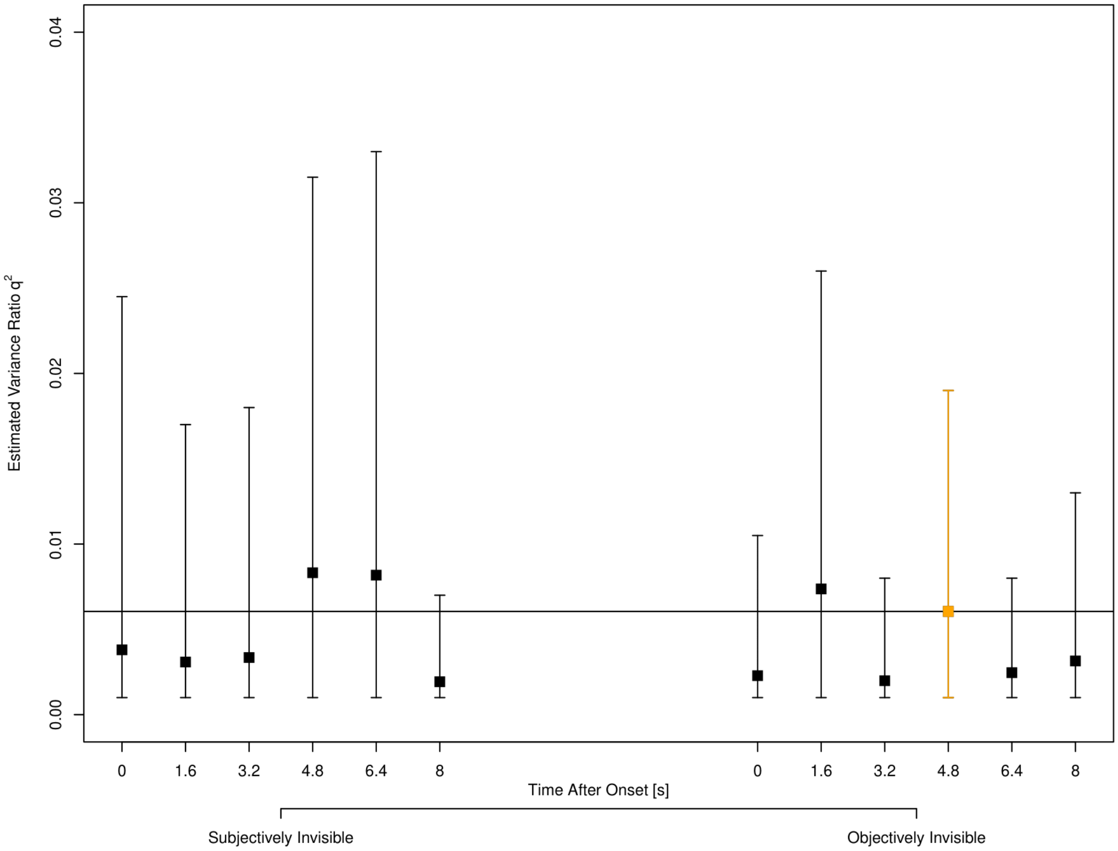

Note. Stein et al. (2021) computed decoding sensitivities d' at different time points after stimulus onset (x-axis) for the subjectively and objectively invisible conditions. Based on these values, estimates for the variance ratio q^2 were derived together with 95% Highest Density Intervals (shown as error bars). The objectively invisible condition at time point 4.8 (colored in orange) yielded the highest decoding sensitivity d'. Therefore, we took the q^2 estimate from that condition for our reanalyses, $q^2 = 0.006$ (horizontal line). On average, q^2 estimates were lower with $q^2 = 0.004$ and $q^2 = 0.005$ in the subjectively and objective invisible condition, respectively. Thus, the q^2 estimate we used is likely an overestimate and increases the chances of finding an Indirect Task Advantage (ITA) in our reanalyses.

3 Original data and reanalysis results

Axelrod et al. (2015) In this study, researchers used continuous flash suppression (CFS) to mask a succession of words, either forming a meaningful or non-meaningful sentence. A localizer with visible words was used to define regions of interest (ROI; LPSTS: left posterior superior temporal sulcus, LMFG: left middle frontal gyrus). Decoding analyses were run on different numbers of voxels (100, 50 and 150). Four sentences were displayed per block, and participants were asked whether these were meaningful sentences at the end of each block. Participants performed distinct direct tasks for the two stimulus types. Both resulted in the same mean accuracy M. We picked the lower standard error (SE) value to follow our benefit-of-the-doubt approach and favor finding an ITA. Only 'guessed' trials were included in the original analysis.

Standard Reasoning: "After each block of either sentences or nonwords participants reported whether they had been aware of even a single word—a procedure which ensured that data analyses were conducted only on blocks judged invisible by participants. To discriminate between neural activity elicited by the 2 conditions we used multivoxel pattern classification analyses (MVPA) focusing on the language network [. . .] The principal goal of our research was to test whether the frontal lobes were involved in any unconscious processing of language." (p. 2160 and 2161)

Table S1: Reported results from Axelrod et al. (2015)

	Indirect measure			Direct measure			Sensitivity Comparison	
	N	K	$d' \pm SE$	N	K	$d' \pm SE$	$d'_{\text{diff}} \pm SE$	CI 95%
LPSTS-100	15	77	0.31 ± 0.10	15	77	0.09 ± 0.10	0.23 ± 0.14	[-0.08, 0.54]
LMFG-100	15	77	0.24 ± 0.07	15	77	0.09 ± 0.10	0.15 ± 0.13	[-0.13, 0.43]
LPSTS-50	15	77	0.32 ± 0.10	15	77	0.09 ± 0.10	0.24 ± 0.15	[-0.08, 0.56]
LMFG-50	15	77	0.21 ± 0.07	15	77	0.09 ± 0.10	0.13 ± 0.13	[-0.14, 0.40]
LPSTS-150	15	77	0.34 ± 0.10	15	77	0.09 ± 0.10	0.25 ± 0.14	[-0.05, 0.56]
LMFG-150	15	77	0.32 ± 0.08	15	77	0.09 ± 0.10	0.24 ± 0.13	[-0.05, 0.52]

Note. LPSTS-n: left posterior superior temporal sulcus (decoding using ROI of n voxels); LMFG: left middle frontal gyrus.

Dehaene et al. (2001). The study consists of two experiments. In E1 the ITA referred to the presence vs. absence of the masked word, while in E2 the ITA referred to a congruency effect (repeated vs. different words). In E1, visibility of words was evaluated using different measures (detection/naming, recognition memory, forced-choice test); for this reanalysis we could only include the forced-choice test. In E1, neural activity was measured using both EEG and fMRI; here we focused on fMRI results. Authors reported Z values at local maxima. We converted these values into t values using quantile mapping (see Methods section).

Standard Reasoning: "In the forced-choice test, on each of 37 trials, a short stream comprising a single masked word was presented. Participants were told about the presence of a hidden word and were asked to select it among two choice words presented left and right of fixation. The success rate of 52.9% did not differ from the 50% value expected by chance [. . .]" (p. 752-753). "Behaviorally, participants again denied seeing the primes and were unable to select them in a two-alternative forced-choice test [...] brain activation was reduced in extrastriate, fusiform and precentral regions similar to those observed in experiment 1. This shows that the repetition suppression phenomenon, which was previously obtained with consciously visible stimuli, can be replicated with unseen masked primes [...]" (p. 755-756).

Table S2: Reported results from Dehaene et al. (2001)

	Indirect measure			Direct measure			Sensitivity Comparison	
	N	K	$d' \pm SE$	N	K	$d' \pm SE$	$d'_{\text{diff}} \pm SE$	CI 95%
LEC E1-LM1	15	150	0.15 ± 0.06	27	37	0.15 ± 0.08	0.00 ± 0.10	[-0.20, 0.20]
LEC E1-LM2	15	150	0.14 ± 0.06	27	37	0.15 ± 0.08	-0.00 ± 0.10	[-0.20, 0.20]
LFG E1-LM1	15	150	0.12 ± 0.05	27	37	0.15 ± 0.08	-0.03 ± 0.10	[-0.22, 0.17]
LFG E1-LM2	15	150	0.12 ± 0.05	27	37	0.15 ± 0.08	-0.03 ± 0.10	[-0.23, 0.17]
LPS E1-LM1	15	150	0.12 ± 0.05	27	37	0.15 ± 0.08	-0.03 ± 0.10	[-0.23, 0.17]
LPS E1-LM2	15	150	0.10 ± 0.05	27	37	0.15 ± 0.08	-0.04 ± 0.10	[-0.24, 0.16]
REC E2-LM1	10	240	0.33 ± 0.10	10	64	0.18 ± 0.10	0.15 ± 0.14	[-0.18, 0.48]
REC E2-LM2	10	240	0.31 ± 0.10	10	64	0.18 ± 0.10	0.13 ± 0.14	[-0.19, 0.45]
LFG E2-CI	10	480	0.17 ± 0.06	10	64	0.18 ± 0.10	-0.01 ± 0.12	[-0.28, 0.25]

Note. LEC: left extrastriate cortex; LFG: left fusiform gyrus; LPS: left precentral sulcus; REC: right extrastriate cortex; E1: experiment 1; E2: experiment 2; LM1: local maximum 1; LM2: local maximum 2; CI: case independent.

Fang et al. (2005). The study reports two experiments. The first experiment does not fit the scope of this reanalysis and was therefore excluded. In E2, tools and faces were rendered invisible using binocular rivalry. In the direct measure, participants were asked to determine in which interval the intact (vs. scrambled) object was presented. In parallel, their BOLD activation in ventral and dorsal ROIs were measured (tools vs. faces; indirect measure). For the indirect task, we estimated the number of trials K based on our best understanding of the methods section and following our benefit-of-the-doubt approach. Note that we used the F value from the main effect incorporating visible and invisible trials. Although we are only interested in the invisible trials (there was no F value for only the invisible condition), the effect is certainly larger in the visible condition and including these trials follows our benefit-of-the-doubt approach. Note also that here the direct measure compares intact vs. scrambled while the indirect measure compares tools vs. faces. This methodological discrepancy between the two measures greatly limits any conclusion that could be made from a confirmed ITA.

Standard Reasoning: "[...] although the subjects could not tell whether the images were faces or tools or even if they were intact, their dorsal cortical neurons still reacted differently to different invisible object images: images of tools induced much stronger BOLD signals in the dorsal ROIs than did images of faces" (p. 1383).

Table S3: Reported results from Fang et al. (2005)

	Indirect measure			Direct measure			Sensitivity Comparison	
	N	K	$d' \pm SE$	N	K	$d' \pm SE$	$d'_{\text{diff}} \pm SE$	CI 95%
Amygdala E2	5	520	0.16 ± 0.10	7	500	-0.02 ± 0.03	0.18 ± 0.11	[-0.08, 0.43]

Note. E2: experiment 2.

Fogelson et al. (2014). In this study, faces and tools were rendered invisible using continuous flash suppression (CFS) or chromatic flicker fusion (CFF). Participants were tested on their ability to discriminate stimuli for both methods (direct measure). MVPA was performed on fMRI data using a linear SVM classifier (indirect measure). We should emphasize that one of the conditions (blinding method = CFF; ROI = fusiform gyrus) almost yielded an ITA (CI 95% = [-0.00044, 0.33251]).

Standard Reasoning: “Behavioral data collected during scanning show that subjects were at chance when guessing stimulus category during both invisible conditions [...] A subset of these regions also showed significant category classification in the absence of stimulus awareness. [...]” (p. 5-6).

Table S4: Reported results from Fogelson et al. (2014)

	Indirect measure			Direct measure			Sensitivity Comparison	
	N	K	$d' \pm SE$	N	K	$d' \pm SE$	$d'_{\text{diff}} \pm SE$	CI 95%
CFS-MOG	17	128	0.10 ± 0.05	17	128	-0.02 ± 0.06	0.11 ± 0.08	[-0.05, 0.27]
CFS-MOLS	17	128	0.09 ± 0.05	17	128	-0.02 ± 0.06	0.10 ± 0.08	[-0.06, 0.27]
CFS-LG	17	128	0.12 ± 0.05	17	128	-0.02 ± 0.06	0.14 ± 0.08	[-0.03, 0.30]
CFF-lg	17	128	0.09 ± 0.05	17	128	-0.03 ± 0.06	0.12 ± 0.08	[-0.04, 0.28]
CFF-FG	17	128	0.14 ± 0.05	17	128	-0.03 ± 0.06	0.17 ± 0.08	[-0.00, 0.33]
CFF-SPS	17	128	0.12 ± 0.05	17	128	-0.03 ± 0.06	0.15 ± 0.08	[-0.02, 0.31]

Note. CFS: continuous flash suppression; CFF: chromatic flicker fusion; MOG: middle occipital gyrus; MOLS: middle occipital and lunate sulci; LG: lingual gyrus; FG: fusiform gyrus; SPS: superior precentral sulcus.

Freeman et al. (2014). Freeman et al. (2012) looked at BOLD signal variations in the amygdala when participants were shown faces with varying degrees of trustworthiness. Real and computer-generated faces with different degrees of facial trustworthiness were presented to participants using backward masking. The study reports two experiments. In E1, the authors used a blocked design to present three levels of facial trustworthiness (low, average, high). In E2, an event-related design allowed the presentation of a continuous range of facial trustworthiness. The two first runs of E2 were subliminal, and the two others were supraliminal. We focused the reanalysis on subliminal runs and values which relied on collapsed conditions (subliminal and supraliminal) were excluded. The authors report the results from one direct measure per experiment (gender discrimination) and from a separate task with different participants (trustworthiness discrimination). We decided to report both. The indirect measure corresponds to difference in BOLD activation in the amygdala between conditions (low trustworthiness vs average). Note that our estimated SE was lower than what authors reported, i.e., it appears that there was more variability in the observed values than we expected. This follows our benefit-of-the-doubt approach, as underestimating the SE will lead to narrower confidence intervals and more confirmed ITAs.

Four analyses out of 16 led to a confirmed ITA, all in experiment 2, namely in the left (d’ difference = 0.16 ± 0.07; 0.19 ± 0.07 for the quadratic effect) and right amygdala (0.17 ± 0.07 and 0.18 ± 0.07 for the quadratic effect). Importantly, we only obtained a confirmed ITA when using the gender discrimination task as a direct measure, not the trustworthiness discrimination task (separate task with different participants). Note that reanalysis results from quadratic analysis are not as straightforward to interpret, but as they were considered as highly valuable evidence for unconscious processing by the authors, we finally decided to include them in the reanalysis.

Standard Reasoning: “d’ overall was quite low (M=0.17, SE=0.11), ensuring that the masked stimuli were below subjects’ awareness. [...] Low-trustworthy targets elicited stronger activation than average-trustworthy targets [...] Thus, regions in the bilateral amygdala exhibited especially strong activation for low-trustworthy faces when presented subliminally.” (p. 10576-10577).

Table S5: Reported results from Freeman et al. (2014)

	Indirect measure			Direct measure			Sensitivity Comparison	
	N	K	$d' \pm SE$	N	K	$d' \pm SE$	$d'_{\text{diff}} \pm SE$	CI 95%
Amygdala E1-Low-GenderDT	19	1440	0.04 ± 0.02	19	144	0.17 ± 0.05	-0.13 ± 0.06	[-0.24, -0.01]
Amygdala E1-Low-TrustDT	19	1440	0.04 ± 0.02	16	211	0.03 ± 0.05	0.01 ± 0.05	[-0.10, 0.13]
R Amygdala E1-Low-GenderDT	19	1440	0.06 ± 0.02	19	144	0.17 ± 0.05	-0.11 ± 0.06	[-0.23, 0.00]
R Amygdala E1-Low-TrustDT	19	1440	0.06 ± 0.02	16	211	0.03 ± 0.05	0.03 ± 0.05	[-0.09, 0.14]
R Amygdala E1-High-GenderDT	19	1440	0.04 ± 0.02	19	144	0.17 ± 0.05	-0.13 ± 0.06	[-0.24, -0.01]
R Amygdala E1-High-TrustDT	19	1440	0.04 ± 0.02	16	211	0.03 ± 0.05	0.01 ± 0.05	[-0.10, 0.13]
R Amygdala E1-quadratic-GenderDT	19	1440	0.06 ± 0.02	19	144	0.17 ± 0.05	-0.11 ± 0.06	[-0.23, 0.01]
R Amygdala E1-quadratic-TrustDT	19	1440	0.06 ± 0.02	16	211	0.03 ± 0.05	0.03 ± 0.05	[-0.08, 0.15]
L Amygdala E2-GenderDT	15	320	0.07 ± 0.04	15	160	-0.09 ± 0.05	0.16 ± 0.07	[0.02, 0.31]
L Amygdala E2-TrustDT	15	320	0.07 ± 0.04	16	211	0.03 ± 0.05	0.04 ± 0.06	[-0.09, 0.17]
R Amygdala E2-GenderDT	15	320	0.08 ± 0.04	15	160	-0.09 ± 0.05	0.17 ± 0.07	[0.02, 0.31]
R Amygdala E2-TrustDT	15	320	0.08 ± 0.04	16	211	0.03 ± 0.05	0.05 ± 0.06	[-0.08, 0.18]
L Amygdala-quadratic-E2-GenderDT	15	320	0.10 ± 0.04	15	160	-0.09 ± 0.05	0.19 ± 0.07	[0.05, 0.34]
L Amygdala-quadratic-E2-TrustDT	15	320	0.10 ± 0.04	16	211	0.03 ± 0.05	0.07 ± 0.06	[-0.06, 0.21]
R Amygdala-quadratic-E2-GenderDT	15	320	0.09 ± 0.04	15	160	-0.09 ± 0.05	0.18 ± 0.07	[0.04, 0.33]
R Amygdala-quadratic-E2-TrustDT	15	320	0.09 ± 0.04	16	211	0.03 ± 0.05	0.06 ± 0.06	[-0.07, 0.20]

Note. GenderDT: gender discrimination (direct task); TrustDT: trustworthiness discrimination (direct task); Low: low vs. average trustworthiness; High: high vs. average trustworthiness; E1: experiment 1; E2: experiment 2; L: left; R: right.

Haynes & Rees (2005). In this study, two experiments are reported. In E1, stimuli are visible and therefore the experiment was excluded from the reanalysis. In E2, Gabor patches are rendered invisible using a masking technique. Participants are asked to report the orientation at the end of each block by pressing on a button (direct measure). A classifier is trained on fMRI data to decode patches' orientation (indirect measure). Note that we estimated decoding values (% accuracy and SE) from Figure 3 because they were not reported in the main text.

Haynes and Rees (2005) reported an accuracy of 50.3% (SE = 0.4%) when four participants attempted to discriminate the orientation of a grating (1170 trials), corresponding to a d' of 0.02 (SE = 0.01). From their Figure 3, we estimated the maximum decoding performance of gratings' orientation in V1 at approximately 58.35% (SE = 2.25%). We considered the data point with the highest decoding accuracy available (100 voxels decoding) in order to follow our benefit-of-the-doubt approach. This decoding performance can be estimated as a d' value of 0.42 (SE = 0.11), resulting in a sensitivity difference of 0.41 (SE = 0.11) and a 95% confidence interval that excludes zero (CI = [0.04-0.77]).

Standard Reasoning: "[...] participants were completely unaware of the orientation of the masked gratings and were at chance performance (50.3 ± 0.4%, s.e.m.) in discriminating their orientation. [...] we found that single volumes could be classified with an accuracy that was significantly above chance for each of the four participants [...] Thus, even when participants' conscious reports indicated that they themselves could not distinguish the orientation of a masked grating, their brain state contained information that could permit such discrimination." (p. 688).

Table S6: Reported results from Hayne & Rees (2005)

	Indirect measure			Direct measure			Sensitivity Comparison	
	N	K	$d' \pm SE$	N	K	$d' \pm SE$	$d'_{\text{diff}} \pm SE$	CI 95%
V1	4	900	0.42 ± 0.11	4	1170	0.02 ± 0.02	0.41 ± 0.11	[0.04, 0.77]

Note. V1: primary visual area.

Kouider et al. (2007). This study reports a subliminal and a supraliminal condition. In the subliminal condition, participants were presented with a masked prime, either unrelated to the target, orthographically similar to the target, or both orthographically similar and homophonic with the target. While participants were performing the task, fMRI data was recorded and the authors tested for a suppression effect, i.e., decrease of BOLD activation in some areas when prime and target were congruent (indirect measure). Immediately after the scanning session, participants' ability to discriminate the prime was tested (direct measure). We did our best to estimate K for the indirect measure based on our understanding of the methods section. Note that we included Z values that were not significant (left and right FEF) or uncorrected (VWFA) because these results nevertheless served the narrative of the reanalyzed study.

Standard Reasoning: "Data from the forced-choice prime identification task were used to evaluate prime visibility. Measures of d' values for each subject confirmed that they were unable to consciously perceive the primes in the subliminal condition [...] the VWFA previously reported in several studies of subliminal priming during reading (Dehaene et al. 2001, 2004; Devlin et al. 2004), also showed a small repetition suppression effect" (p. 2022-2023).

Table S7: Reported results from Kouider et al. (2007)

	Indirect measure			Direct measure			Sensitivity Comparison	
	N	K	$d' \pm SE$	N	K	$d' \pm SE$	$d'_{\text{diff}} \pm SE$	CI 95%
LFEF	15	448	0.21 ± 0.05	15	180	0.19 ± 0.05	0.02 ± 0.08	[-0.14, 0.18]
RFEF	15	448	0.17 ± 0.05	15	180	0.19 ± 0.05	-0.02 ± 0.07	[-0.17, 0.14]
VWFA	15	448	0.14 ± 0.04	15	180	0.19 ± 0.05	-0.05 ± 0.07	[-0.20, 0.09]

Note. LFEF: left frontal eye field; RFEF: right frontal eye field; VWFA: visual word form area.

Kouider et al. (2009). In this study, participants were presented with familiar and unfamiliar faces and were asked to perform a fame-judgment task. Target faces were preceded by a masked face, either the same picture (same-view), the same face but viewed from a different angle (cross-view), or a different face. BOLD variations were measured and repetition suppression effects were obtained (indirect measure 1). In another phase of the experiment, an ANOVA was performed on four selected face-responsive ROIs (indirect measure 2). For this second indirect measure, we interpreted N to be 13 instead of 16 to be in agreement with our benefit-of-the-doubt approach. In a separate task, participants were asked to identify if the prime face was familiar or unfamiliar (direct measure). Note that data from another direct measure was available but we decided to reanalyze the fame-judgment task, as it was performed with the same participants and as fame was the feature of interest of the main experiment. Moreover, the prime fame-judgment task resulted in a lower d' than the other task (d'= 0.34). Selecting the fame-judgment task as a direct measure therefore follows our benefit-of-the-doubt approach.

Standard Reasoning: "The forced-choice fame-judgment task on the primes confirmed that our

masking method rendered the primes largely invisible, as performance was close to chance [...]" (p. 16). [. . .] we found evidence of repetition-related hemodynamic response decreases (i.e., repetition suppression) in several regions of the occipitotemporal cortex. [. . .] These data provide evidence that face processing can occur in face-processing regions within the ventral visual stream in the absence of perceptual awareness." (p. 18).

Table S8: Reported results from Kouider et al. (2009)

	Indirect measure			Direct measure			Sensitivity Comparison	
	N	K	$d' \pm SE$	N	K	$d' \pm SE$	$d'_{\text{diff}} \pm SE$	CI 95%
RSTG&S	16	480	0.18 ± 0.05	16	64	0.16 ± 0.08	0.02 ± 0.09	[-0.18, 0.22]
RSTG&S-2	16	480	0.15 ± 0.04	16	64	0.16 ± 0.08	-0.01 ± 0.09	[-0.21, 0.18]
LLOC&PMTG	16	480	0.17 ± 0.05	16	64	0.16 ± 0.08	0.01 ± 0.09	[-0.18, 0.21]
LLOC&PMTG-2	16	480	0.16 ± 0.04	16	64	0.16 ± 0.08	0.00 ± 0.09	[-0.19, 0.20]
LLOC&PMTG-3	16	480	0.16 ± 0.04	16	64	0.16 ± 0.08	-0.00 ± 0.09	[-0.20, 0.19]
LMFG	16	480	0.17 ± 0.04	16	64	0.16 ± 0.08	0.01 ± 0.09	[-0.19, 0.20]
RSTG	16	480	0.15 ± 0.04	16	64	0.16 ± 0.08	-0.01 ± 0.09	[-0.20, 0.18]
RPMTG	16	480	0.14 ± 0.04	16	64	0.16 ± 0.08	-0.02 ± 0.09	[-0.21, 0.17]
RPMTG-2	16	480	0.13 ± 0.04	16	64	0.16 ± 0.08	-0.03 ± 0.09	[-0.22, 0.16]
4FROIS Global priming	13	480	0.09 ± 0.04	16	64	0.16 ± 0.08	-0.07 ± 0.09	[-0.26, 0.12]
4FROIS Same	13	320	0.09 ± 0.04	16	64	0.16 ± 0.08	-0.07 ± 0.09	[-0.26, 0.13]
4FROIS Cross	13	320	0.08 ± 0.04	16	64	0.16 ± 0.08	-0.08 ± 0.09	[-0.27, 0.12]

Note. RSTG&S: right superior temporal gyrus and sulcus; LLOC&PMTG: left lateral occipital complex and posterior middle temporal gyrus; LMFG: left mid-fusiform gyrus; RSTG: right superior temporal gyrus; RPMTG: right posterior middle temporal gyrus; 4FROIS: 4 functional regions of interest they selected for the omnibus ANOVA; Same: same-view faces; Cross: cross–view faces.

Kouider et al. (2016). In this study, twenty participants were alternately asked to detect either faces, flowers or watches, meaning that these stimuli could be either task-relevant or task-irrelevant. Stimuli were masked and presentation duration was either 200ms (visible) or 33ms (invisible). Ten different participants performed a discrimination task outside of the scanner to assess stimulus visibility (direct measure). Note that stimulus awareness was also tested during the main experiment, but the separate task was considered a more valid way to assess awareness by the authors. An ANOVA (stimulus category x task relevance) and t-test analyses were performed on the BOLD activation differences in FFA between the different conditions (indirect measure).

Standard Reasoning: "[...] the stimuli in the invisible conditions could not be discriminated not only when responding solely to the target category (d' = 0.061, t < 1), but also when performing a forced-choice on each trial (d' = -0.013, t < 1)." (p. 4). "Further analysis revealed that FFA responses to faces were enhanced during face relevance blocks relative to alternative relevance blocks [...] the FFA responded more to invisible faces than to the other invisible objects in the face detection blocks [...] the activity for invisible faces was amplified relative to either invisible alternative objects (t(19) = 2.246, P = 0.037) or to invisible control objects (t(19) = 2.237, P = 0.038)." (p. 5) "Our study goes further by revealing that selective attention, in the absence of awareness, involves not only response enhancement for task-relevant information, but also the active filtering of distracting information in visual cortex [...] The current study provides further evidence for the flexibility of non-conscious perceptual processes, by showing that sensory regions can in turn be modulated by task relevance." (p. 7).

Table S9: Reported results from Kouider et al. (2016)

	Indirect measure			Direct measure			Sensitivity Comparison	
	N	K	$d' \pm SE$	N	K	$d' \pm SE$	$d'_{\text{diff}} \pm SE$	CI 95%
FFA Faces (FD vs. AD)	20	360	0.08 ± 0.03	10	180	-0.01 ± 0.06	0.09 ± 0.07	[-0.07, 0.25]
FFA Faces vs. Objects (FD)	20	180	0.10 ± 0.04	10	180	-0.01 ± 0.06	0.11 ± 0.08	[-0.06, 0.28]
FFA Objects vs Faces (AD)	20	180	0.11 ± 0.04	10	180	-0.01 ± 0.06	0.13 ± 0.08	[-0.05, 0.30]
FFA Faces vs. Alternative (FD)	20	120	0.10 ± 0.05	10	180	-0.01 ± 0.06	0.11 ± 0.08	[-0.07, 0.29]
FFA Faces vs. Control (FD)	20	120	0.10 ± 0.05	10	180	-0.01 ± 0.06	0.11 ± 0.08	[-0.07, 0.29]
FFA Faces vs. Control (AD)	20	120	0.13 ± 0.05	10	180	-0.01 ± 0.06	0.14 ± 0.08	[-0.04, 0.33]
FFA Faces vs. Mask-only	20	120	0.13 ± 0.05	10	180	-0.01 ± 0.06	0.14 ± 0.08	[-0.04, 0.33]

Note. FFA: fusiform face area; FD: face detection task; AD: alternative object detection task.

Moutoussis & Zeki (2002). In this study, the authors use a method called dichoptic fusion to render stimuli (houses and faces) invisible. Visibility was assessed before scanning using a discrimination task (direct measure). Crucially, here we reanalysed the t threshold for single-voxel significance as defined by the authors ($t = 3.12$; indirect measure). It is important to stress that some results from which the values were not reported in the article might have been non-negligibly above this threshold, potentially yielding an ITA. However, results based on this significance threshold reported by the authors are not sufficient to support their claims.

Standard Reasoning: "[...] in the opposite conditions, the 2AFC gave an average performance of 52.7% (SD = 4.2); 4/7 subjects scored above 50%, the highest score being 59.4%." "The resultant parameter estimates for each regressor at each voxel were compared by using t tests to determine whether significant activation resulting from a comparison of conditions had occurred. [...] Although the extent of activation was not as widespread as with perceived stimuli, it still is surprising that many "higher," binocularly driven areas of the brain are activated by these invisible stimuli when compared with the (perceptually equivalent) uniform controls." (p. 9528-9529).

Table S10: Reported results from Moutoussis & Zeki (2002)

	Indirect measure			Direct measure			Sensitivity Comparison	
	N	K	$d' \pm SE$	N	K	$d' \pm SE$	$d'_{\text{diff}} \pm SE$	CI 95%
Significance threshold	7	122	0.20 ± 0.11	7	32	0.14 ± 0.08	0.07 ± 0.13	[-0.26, 0.39]

Schurger et al. (2010). In this study, participants were presented with face and house stimuli which were masked using dichoptic-color masking. Participants' awareness was tested on each trial using a discrimination task (direct measure). A Gaussian naïve Bayes classifier was trained on fMRI data (temporal lobes; indirect measure). Note that the authors also used wagering (confidence in one's answer) as a collateral index of one's awareness.

Standard Reasoning: "For visible stimuli, performance was at or near 100% correct for all 12 subjects, and all wagers were high. For invisible stimuli, task performance was only marginally different from chance (54 ± 2.5[SEM]% correct; $P < 0.06$, one-tailed t test) [...] We used multivariate pattern analysis to ascertain how the encoding of perceptual information differs depending on whether or not that information is present in subjective experience (17). Thus, in our analyses we focused specifically on the patterns of activation corresponding to the perceptual information of which the subject was or was not aware: the category of the object." (p. 97).

Table S11: Reported results from Schurger et al. (2010)

	Indirect measure			Direct measure			Sensitivity Comparison	
	N	K	$d' \pm SE$	N	K	$d' \pm SE$	$d'_{\text{diff}} \pm SE$	CI 95%
Temporal lobes	12	288	0.40 ± 0.15	12	288	0.20 ± 0.12	0.20 ± 0.20	[-0.24, 0.64]

Stein et al. (2021). Participants were asked to discriminate masked house and face stimuli after each trial, while lying in the scanner (direct measure). The authors examined BOLD activity patterns in four regions of interest (V1, LOC, OFA/OPA, FFA/PPA; indirect measure).

Standard Reasoning: "Importantly, in obj-inv trials, discrimination performance (M = 0.02, SD = 0.25) did not differ significantly from chance (Fig 1E), with moderate evidence for the null hypothesis of chance level discrimination [...]" (p. 5). "Activity patterns in LOC discriminated between faces and houses with above-chance accuracy in all visibility conditions [...] and also in obj-inv (t(42) = 3.28, p = 0.001, dz = 0.50, BF+0 = 31.19). Thus, LOC contained category information for both subjectively and objectively invisible stimuli." (p. 6).

Table S12: Reported results from Stein et al. (2021)

	Indirect measure			Direct measure			Sensitivity Comparison	
	N	K	$d' \pm SE$	N	K	$d' \pm SE$	$d'_{\text{diff}} \pm SE$	CI 95%
V1	43	200	0.06 ± 0.03	43	200	0.02 ± 0.03	0.04 ± 0.04	[-0.04, 0.12]
LOC	43	200	0.08 ± 0.03	43	200	0.02 ± 0.03	0.06 ± 0.04	[-0.02, 0.14]
OFA/OPA	43	200	0.07 ± 0.03	43	200	0.02 ± 0.03	0.05 ± 0.04	[-0.03, 0.12]

Note. V1: primary visual area; LOC: lateral occipital complex; OFA/OPA: occipital face area/occipital place area.

Sterzer et al. (2008). In this study, MVPA was used to decode house and face stimuli rendered invisible using continuous flash suppression (CFS). Participants' awareness was tested using both a discrimination task (direct measure) and a subjective assessment. Classification was performed on the fMRI data from two main ROIs (FFA and PPA) using linear support vector machines (indirect measure). 4,1% of the trials were excluded because they were subjectively visible. One run was excluded because the participant reported being unable to binocularly fuse the stimuli due to sleepiness.

Standard Reasoning: "Discrimination of invisible faces and houses was tested in a 2-alternative forced-choice task performed directly after each block; performance was at chance level in all participants (average d'= -0.05 ± 0.14 SEM, p = 0.71, one-sample t-test). [...] Strikingly, prediction accuracy for invisible stimuli (where univariate analyses had failed to show differences between activity evoked by face and house stimuli) was also significantly above chance level (FFA: 58.8% ± 2.3 SEM, t(4) = 3.8, p = 0.019; PPA: 62.5% ± 3.3 SEM, t(4) = 3.8, p = 0.019; FFA + PPA: 63.5% ± 3.7 SEM, t(4) = 3.6, p = 0.022)." (p. 6-7). "Our data demonstrate that activity patterns in the FFA and the PPA differentiate two categories of object stimuli (faces and houses) even when the stimuli are rendered invisible by interocular suppression." (p. 9).

Table S13: Reported results from Sterzer et al. (2008)

	Indirect measure			Direct measure			Sensitivity Comparison	
	N	K	$d' \pm SE$	N	K	$d' \pm SE$	$d'_{\text{diff}} \pm SE$	CI 95%
FFA	5	77	0.44 ± 0.11	5	77	-0.05 ± 0.13	0.49 ± 0.18	[0.01, 0.98]
PPA	5	77	0.64 ± 0.16	5	77	-0.05 ± 0.13	0.69 ± 0.21	[0.10, 1.27]
FFA&PPA	5	77	0.69 ± 0.18	5	77	-0.05 ± 0.13	0.74 ± 0.23	[0.11, 1.37]

Note. FFA: fusiform face area; PPA: parahippocampal place area.

Ulrich & Kiefer (2016). Participants were presented with white geometrical shapes (circle, diamond, square, ellipsoid). Two of these shapes were mapped to the left index finger (circle and diamond) and the two others to the right index finger (square and ellipsoid), or vice versa. These target shapes were preceded by either congruent or incongruent primes that were rendered invisible using a forward and a backward line pattern mask. Variations in brain activity were measured using fMRI (indirect task). Both congruent (5.7%) and incongruent trials (6%) were excluded because of being either incorrect or missing. After scanning, participants' awareness of the prime was assessed inside the scanner using a discrimination task (direct task). Note that part of the claim of this study relies on functional connectivity analyses that could not be included in the reanalysis.

Standard Reasoning: "The d′ measure of prime visibility (Green and Swets 1966) was 0.03 on average (SD = 0.39) and did not significantly deviate from zero (t(30) = 0.43, P = 0.670)." (p.2475). "[...] analyses revealed lower brain activity for congruent (CON) than incongruent (INC) trials in ventrolateral and dorsomedial frontal and inferior parietal brain regions as well as in the basal ganglia. The present study thereby confirms and extends earlier findings by demonstrating an involvement of the visuomotor network in subliminal visuomotor processing [...]" (p.2476).

Table S14: Reported results from Ulrich & Kiefer (2016)

	Indirect measure			Direct measure			Sensitivity Comparison	
	N	K	$d' \pm SE$	N	K	$d' \pm SE$	$d'_{\text{diff}} \pm SE$	CI 95%
RIPL	31	120	0.19 ± 0.04	31	48	0.03 ± 0.07	0.16 ± 0.08	[-0.00, 0.33]
RMSFG	31	120	0.19 ± 0.04	31	48	0.03 ± 0.07	0.16 ± 0.08	[-0.00, 0.32]
RIFG	31	120	0.18 ± 0.04	31	48	0.03 ± 0.07	0.15 ± 0.08	[-0.01, 0.31]
LIPL	31	120	0.18 ± 0.04	31	48	0.03 ± 0.07	0.15 ± 0.08	[-0.01, 0.31]
RCN	31	120	0.17 ± 0.04	31	48	0.03 ± 0.07	0.14 ± 0.08	[-0.02, 0.30]
LIFG	31	120	0.17 ± 0.04	31	48	0.03 ± 0.07	0.14 ± 0.08	[-0.02, 0.30]

Note. RIPL: right inferior parietal lobule; RMSFG: right medial superior frontal gyrus; RIFG: right inferior frontal gyrus; LIPL: left inferior parietal lobule; RCN: right caudate nucleus; LIFG: left inferior frontal gyrus.

van Gaal et al. (2010). Participants performed a go/no-go task with no-go signals rendered invisible using metacontrast masking. Neural activity was measured using fMRI while participants were performing the go/no-go task (indirect measure). To assess their awareness of the no-go signal, participants were asked to perform a discrimination task while still lying in the scanner and a d-prime value was computed (direct measure). Four participants who performed better than chance at the discrimination task were excluded from further analysis (regression to the mean).

Standard Reasoning: "The combination of these factors effectively rendered the participants incapable of perceiving the square/diamond, as evidenced by chance-level performance on a two-choice discrimination task administered after the experiment [...]" (p. 4144). "To examine the acti-

vation related to the unconscious initiation of inhibitory control, we contrasted responded, strongly masked no-go trials with responded, strongly masked go trials."(p. 4146). "In a go/no-go paradigm, we masked no-go signals to the point that they could no longer be detected to investigate the depth of processing of strongly masked (unconscious) no-go signals in the human brain. Strongly masked no-go signals were observed to activate brain regions central to networks that have been associated with conscious response inhibition, namely the IFC and the pre-SMA." (p. 4147).

Table S15: Reported results from van Gaal et al. (2010)

	Indirect measure			Direct measure			Sensitivity Comparison	
	N	K	$d' \pm SE$	N	K	$d' \pm SE$	$d'_{\text{diff}} \pm SE$	CI 95%
RIFC	20	240	0.17 ± 0.05	20	48	0.12 ± 0.08	0.05 ± 0.09	[-0.14, 0.25]
LIFC	20	240	0.15 ± 0.04	20	48	0.12 ± 0.08	0.03 ± 0.09	[-0.16, 0.22]
pre-SMA	20	240	0.14 ± 0.04	20	48	0.12 ± 0.08	0.02 ± 0.09	[-0.17, 0.21]

Note. RIFC: right inferior frontal cortex; LIFC: left inferior frontal cortex; Pre-SMA: pre-supplementary motor area.

Yang et al. (2012). In this study, masked fearful and neutral face stimuli were presented to participants during an encoding phase. During the retrieving phase, half the faces had the same valence as during the encoding and half not. After the main experiment, participants were asked to perform a discrimination task during a post-awareness assessment, outside of the scanner (direct measure). BOLD variations in the amygdala and other brain regions were measured during the encoding and retrieving phases (indirect measure). The specificity of this study compared to the others included in the reanalysis is that two groups of participants were constituted based on awareness scores (regression to the mean). We focused the reanalysis on data collected from the unaware group.

Standard Reasoning: "[...] the unaware participants (N=13) had a chance level in detecting the faces (p >0.1). [...] for unaware participants, fearful faces (vs. neutral) produced stronger activation in the right amygdala (20, 5, 8, t (12) = 6.39) and the right pulvinar (6, 11, 6, t (12) = 4.96) [...] the amygdala activation was different for unaware and aware participants during encoding, but similar for the two groups during retrieval." (p. 5-6).

Table S16: Reported results from Yang et al. (2012)

	Indirect measure			Direct measure			Sensitivity Comparison	
	N	K	$d' \pm SE$	N	K	$d' \pm SE$	$d'_{\text{diff}} \pm SE$	CI 95%
R Amygdala	13	240	0.25 ± 0.07	13	40	0.09 ± 0.11	0.16 ± 0.13	[-0.13, 0.45]
R Pulvinar	13	240	0.19 ± 0.06	13	40	0.09 ± 0.11	0.10 ± 0.13	[-0.17, 0.38]

Note. R Amygdala: right amygdala; R Pulvinar: right pulvinar.
